# Establishing an Appropriate Pressure for the Transparent Disc Method to Distinguish Early Pressure Injury and Blanchable Erythema

**DOI:** 10.3390/diagnostics12051075

**Published:** 2022-04-25

**Authors:** Lu Chen, Yuan Yuan, En Takashi, Akio Kamijo, Jingyan Liang, Jianglin Fan

**Affiliations:** 1 Division of Basic & Clinical Medicine, Faculty of Nursing, Nagano College of Nursing, Komagane 399-4117, Japan; cl591274839@163.com (L.C.); 006707@yzu.edu.cn (Y.Y.); a.kamijo@nagano-nurs.ac.jp (A.K.); 2 Department of Molecular Pathology, Faculty of Medicine, Graduate School of Medical Sciences, University of Yamanashi, 1110 Shimokato, Chuo 409-3898, Japan; 3 School of Nursing, Yangzhou University, Yangzhou 225001, China; 4 Institute of Translational Medicine, Medical College, Yangzhou University, Yangzhou 225001, China; jyliang@yzu.edu.cn

**Keywords:** early pressure injury, blanchable erythema, appropriate pressure, transparent disc method

## Abstract

Background: Non-blanchable erythema is used as a diagnostic indicator for stage 1 pressure injury (early PI); it is distinguished from blanchable erythema (BE) by the application of “light pressing”. Considering the low of the accuracy of the degree of pressure applied, it is difficult to use this method in clinical settings. Methods: We constructed models of BE and early PI in order to determine the most appropriate pressure values using the transparent disc method. We observed erythema by using a Dermo-camera to quantify the gray and a* values of the wound area along with a spectrophotometer. Results: BE started to fade at 50 mmHg, while the gray values became statistically significant when the pressure was increased to 100 mmHg (*p* < 0.05). However, erythema remained even when the pressure was increased to 150 mmHg soon after decompression. By contrast, the early PI was showed to be non-blanchable for the longest time under a pressure of 150 mmHg, but by 18 h it had decreased and the erythema faded more obviously after applying pressure. Conclusions: We proposed that a pressure of 50–100 mmHg was more appropriate for light pressure, but this may vary when different instruments are used. Variations may occur in either BE or early PI, therefore, careful attention should be paid during observations.

## 1. Introduction

Pressure injury (PI) is a refractory skin disease that seriously affects the health of patients and threatens lives [1]. Therefore, its early diagnosis and treatment are very important. With the development of medical technology, early treatment has become possible in clinical work. In this aspect, the National Pressure Injury Advisory Panel (NPIAP) changed the terminology of “Pressure Ulcer” to “Pressure Injury” in 2016, to emphasize the existence of early PI before ulcer formation [2]. Stage 1 is the earliest stage of PI (early PI), while non-blanchable erythema is the diagnosis indicator in the transparent disc method, and this condition is mainly caused by intradermal hemorrhage and can be distinguished from the BE caused by hyperemia [3].

Although the transparent disc method is not an ideal detection method, and may even cause secondary damage due to excessive pressure, there is no better alternative method at present. In transparent disc method, fingers or transparent disk method are used to gently press the area of erythema. This method is widely utilized in clinical settings because of its convenience [4]. However, the process of applying light pressure to the erythema is not clearly defined. This affects the results of inspections, as the pressure exerted by the estimator will vary from person to person, leading to the omission of some epidemiological statistics. In addition, the prevalence and incidence found in these statistical studies were lower than the actual prevalence of PI [5]. In clinical settings, although a pressure of 150 mmHg was reported, this was just based on clinical experience and lacked sufficient evidence [6]. For this reason, the appropriate pressure to use in the transparent disc method should be scientifically verified and determined. Furthermore, for either early PI or BE, a process of the development or disappearance of the wound area occurs. During this period, whether the changes in redness or erythema will affect the results of diagnosis via the transparent disc method is also an issue that should be explored.

In the present study, we verified the uncertainty of the degree of ‘light pressure’ through human observations. Moreover, animal models of BE and early PI were established. A pressure attachment device was made to simulate the pressure of the transparent disc method, and a spectrophotometer and Dermo-camera were used to measure and observe the fading of erythema under different pressures throughout the process and determine the appropriate pressure in order to improve the results of the transparent disc method.

## 2. Materials and Methods

### 2.1. Animals

Twelve-week -old male hairless rats (HWY/Slc, SLC, Inc., Shizuoka, Japan) were used in this experiment. The animals were given a standard chow diet with free access to water and kept in a 12-h light/dark cycle throughout the entire experiment. The protocol was approved by the Committee on the Ethics of Animal Experiments of the Nagano College of Nursing (No. 2021-4).

### 2.2. Generation of BE and Early PI Models

Two rat PI models representing BE and early PI were constructed and analyzed according to the method described in a previous study with slight modifications [7]. In brief, hairless rats were anesthetized with isoflurane inhalation (import: 4–5%; maintenance: 2–3%) (Meiji Seika Pharma Co., Ltd., Tokyo, Japan). For the generation of BE models, the symmetrical part of the left or right back skin was lifted gently and then pressed for 45 min using two circular neodymium magnets (10 mm in diameter × 4 mm in thickness) (Niroku Seisakusho Co., Ltd., Kobe, Japan) with an attraction of 440 mmHg to exert pressure. Afterwards, we started to observe the changes in erythema at the start of the experiment, and 5, 10, 20, 30 min and 60 min after decompression. For the generation of early PI models, the time for which the pressure was applied lasted for 3 h and 50 min. Afterwards, we observed the changes in erythema at 0.5, 6, 12, 18, 24, 30, 60 and 96 h after decompression. Preliminary experimental results showed that erythema become blanchable after 45 min of compression and disappears within an hour. Spectrophotometric analysis was then performed on the left side, while a Dermo-camera was used on the right side of the rats to prevent the results being affected by indentation at a short time interval.

### 2.3. Macroscopic Observations

After erythemas were formed, the magnets were moved. After decompression, a digital camera (Optio WG-3 PENTAX) was used to take photos of the wounds at the start of the experiment, and 5, 10, 20, 30 and 60 min of BE. A camera (Optio WG-3 PENTAX) was used to take photos of the early PI wounds at 0.5, 6, 12, 18, 24, 30, 60 and 96 h after decompression. The distance between the skin wounds and the camera was fixed at 20 cm. The optical magnification used was 4×. The normal skin before the construction of the rat model was designated as the control.

### 2.4. Establishing Pressure of Transparent Disc Method

To simulate the transparent disc method, we fixed a Dermo-camera on a digital force gauge (DST series, Imada Co., Toyohashi, Japan). The wound area and surrounding skin of the rats were placed on a round platform (2 cm in diameter) and then subjected to different levels of pressure. Pressure was regulated with an electric stand (MX500N, Imada Co., Toyohashi, Japan). The pressure was set in the range of blank to150 mmHg. The rats were anesthetized as mentioned above to maintain sedation and thus ensure the accuracy of the pressure applied. The camera’s mode was set to the animation DEMO to capture the whole process. Afterward, photos were obtained at different pressures of blank, 25, 50, 75, 100, 125 and 150 mmHg. The maximum pressure was set to 150 mmHg based on our pre-experiment and previous clinical studies [6].

Under 150 mmHg pressure, the erythema color was close to white. Then, we used the Image J software to measure the gray values in order to evaluate the fading of erythema under the pressure applied in the simulated transparent disk method.

### 2.5. Spectrophotometry Analysis

In this analysis, a spectrophotometer (NF555 NIPPON DENSHOKU, Tokyo, Japan) was used instead of the Dermo-camera, and other pressure and pressure recording equipment were the same as those listed above. The spectrophotometer was pressed on the skin erythema, and the light reflectivity of different wavelengths was recorded. The a* values were obtained to assess changes in skin erythema as in the previous study [8].

### 2.6. Statistical Analysis

All the data are expressed as mean ± SEM. Statistical tests were examined using the GraphPad Prism 7.0 software (GraphPad Software, San Diego, CA, USA). Each data set was first assessed for normality using the Shapiro-Wilk test. An unpaired-sample t test was used to analyze nonparametric distributed data. Statistical significance was set as less than 0.05.

## 3. Results

**Figure 1:**
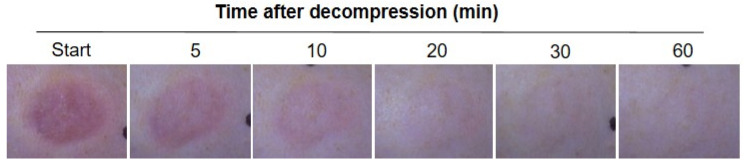
Representative macroscopic observation of blanchable erythema. The progress of erythema after decompression showed at the start and, 5, 10, 20, 30 and 60 min.

To establish the appropriate pressure to use in the transparent disc method for BE animal models, we first made continuous observations of skin BE color changes based on serial digital pictures, as shown in Figure 1. After decompression, erythema was clearly observed at the starting point and remained visible until 5 min. With the passing of time, the erythema gradually faded over 10 min after decompression. As shown in Figure 1, the erythema completely disappeared at 1 h.

### 3.1. Establish the Appropriate Pressure for the Transparent Disc Method 

**Figure 2:**
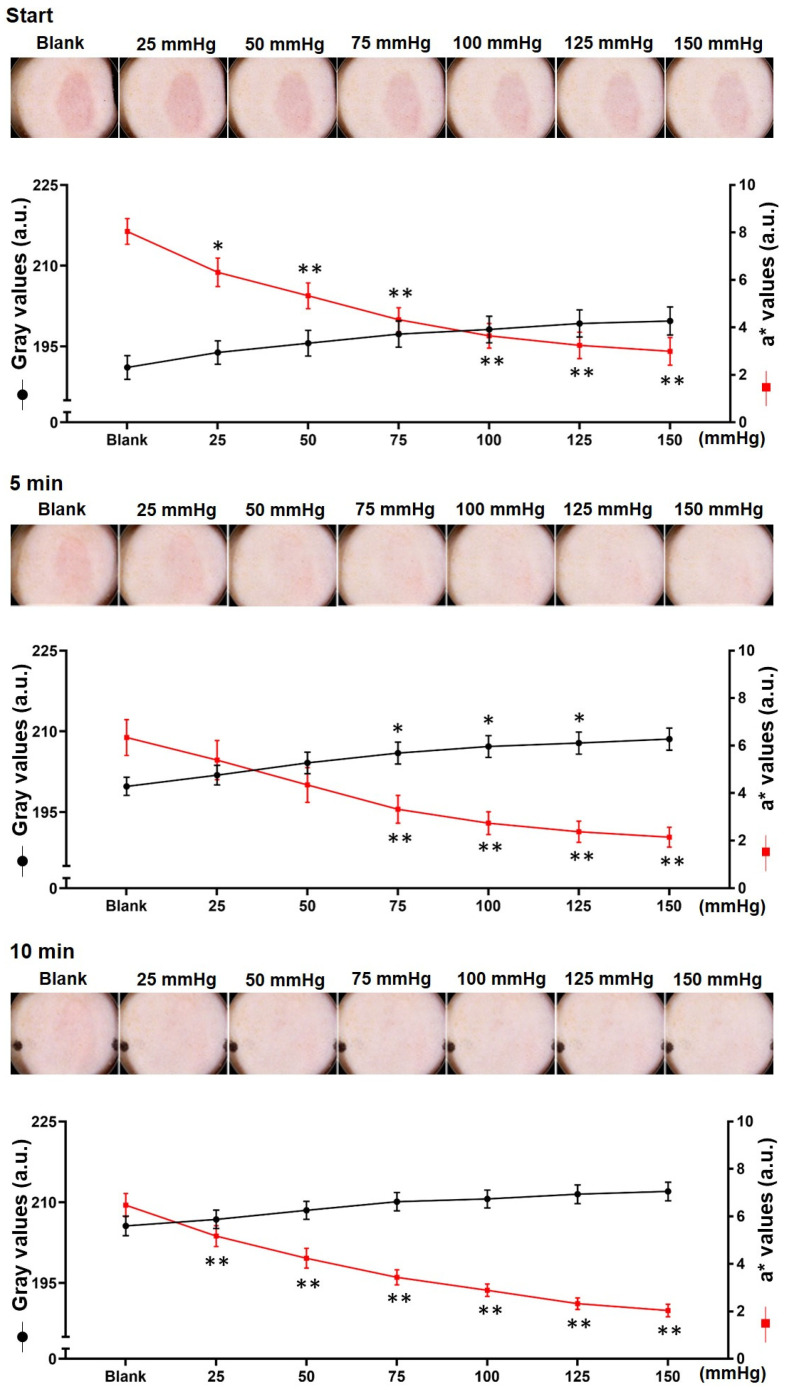
Macroscopic observations and quantification of blanchable erythema. Macroscopic observations of the loaded area at the start (**top panel**) and, 5 (**middle panel**) and 10 min (**bottom panel**) after decompression. The pressure range of the transparent disc method was from blank to 150 mmHg. Representative pictures are shown at the top of each panel. The blanchable erythema area (gray value) was quantified using the Image J software and the erythema index was regarded as a* value measured by the spectrophotometer. (bottom; n = 8 for each group; * *p* < 0.05, ** *p* < 0.01 vs. blank via 1-way ANOVA and Dunnett’s post hoc test). Black color indicates the gray value; red color indicates the erythema index.

After decompression, we took pictures of BE using the Dermo-camera at the start and after, 5 and 10 min (Figure 2, top panel). After decompression, the residual erythema was still obvious despite the applied pressure of 150 mmHg, and the surrounding skin showed obvious signs of fading. The erythema faded significantly when a pressure of 50 mmHg was applied at 5 and 10 min after decompression, and the erythema disappeared under the applied pressure of 100 mmHg.

The gray and a* values of the macroscopic images of erythema were measured (Figure 2, bottom panel).

The gray value of erythema tended to increase three times: from 191.1 ± 5.8 to 199.7 ± 6.9 at the start, from 199.8 ± 4.4 to 208.6 ± 5.4 at 5 min, and a final significant increase at 100 mmHg (*p* < 0.01 vs. blank). At 10 min after decompression, it increased from 205.6 ± 4.8 to 212 ± 4.6.

The a* value of BE decreased from 8 ± 1.4 to 3 ± 1.5 at the start, from 6.3 ± 2.0 to 2.1 ± 1.1 at 5 min, and from 6.5 ± 1.3 to 2.0 ± 0.7 at 10 min. When the pressure was 50 mmHg, the three time periods were remarkably reduced (*p* < 0.01 vs. blank).

As shown in Figure 2, bottom, the erythema of BE disappeared rapidly after decompression, while the gray value increased from 191.1 ± 5.8 at the start after decompression to 199.8 ± 4.4 at 5 min (*p* < 0.05 vs. start), and increased even more from 10 min to approximately 205.6 ± 4.8 (*p* < 0.01 vs. start).

### 3.2. Analysis of Pressure for Early PI as Determined Using Transparent Disc Method 

**Figure 3:**
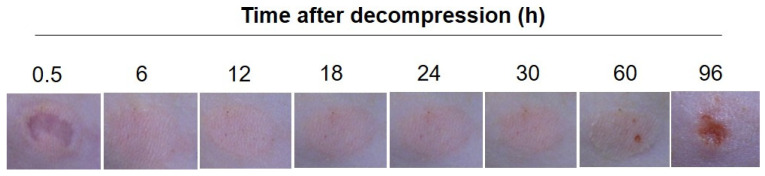
Representative macroscopic observation of the early PI. Macroscopic observations of the loaded area were taken 0.5, 6, 12, 18, 24, 30, 60 and 96 h after decompression.

The erythema of PI was visible until 24 h after decompression. A slight increase or decrease was observed during the period. At 30 h after decompression, the erythema began to show punctate erosion and an ulcer formed by 96 h (Figure 3). 

**Figure 4:**
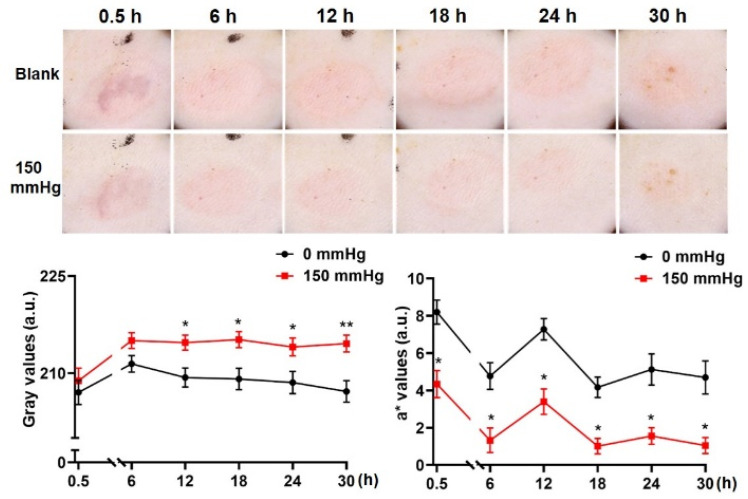
Macroscopic observation and quantification of the early PI. Macroscopic observations of the loaded area were taken 0.5, 6, 12, 18, 24 and 30 h after decompression. The pressure range of the transparent disc method went from blank and 150 mmHg. Representative pictures are shown in the top panel. The loaded area (gray value) was quantified using the Image J software and the erythema index was regarded as the a* value measured by the spectrophotometer. (**bottom**; n = 8 for each group; * *p* < 0.05, ** *p* < 0.01 vs. blank via 1-way ANOVA and Dunnett’s post hoc test).

When pressure was applied, non-blanchable erythema was observed at 0.5, 6, 12, 24 and 30 h after decompression, although all instances were alleviated. However, the erythema faded significantly 18 h after decompression (Figure 4).

When the pressure was applied, the gray value of erythema was respectively decompressed from 207 ± 4.9 (0.5 h) to, 211.4 ± 3.4 (6 h), 209.3 ± 3.9 (12 h), 209.1 ± 4.4 (18 h), 208.6 ± 4.5 (24 h) and 207.2 ± 4.4 (30 h) and increased to 208.8 ± 5.1 (0.5 h), 215 ± 3.2 (6 h), 214.7 ± 3.0 (12 h, *p* < 0.05 vs. blank), 215.2 ± 3.3 (18 h, *p* < 0.05 vs. blank), 214.1 ± 3.6 (24 h, *p* < 0.05 vs. blank) and 214.6 ± 3.4 (30 h, *p* < 0.01 vs. blank).

The a* value ranged from 8.2 ± 1.7 (0.5 h) to, 4.8 ± 1.9 (6 h), 7.3 ± 1.5 (12 h, *p* < 0.05 vs. 0 mmHg), 4.2 ± 1.5 (18 h), 5.1 ± 2.2 (24 h) and 4.7 ± 2.3 (30 h), then reduced to 4.3 ± 1.9 (0.5 h, *p* < 0.05 vs. blank), 1.3 ± 1.8 (6 h, *p* < 0.05 vs. blank), 3.4 ± 1.8 (12 h, *p* < 0.05 vs. blank), 1.0 ± 1.1 (18 h, *p* < 0.05 vs. blank), 1.6 ± 1.2 (24 h, *p* < 0.05 vs. blank) and 1.1 ± 1.1 (30 h, *p* < 0.05 vs. blank) (Figure 4, bottom).

### 3.3. Observation of the Variations of Erythema When the Transparent Disc Was Pressed

**Figure 5:**
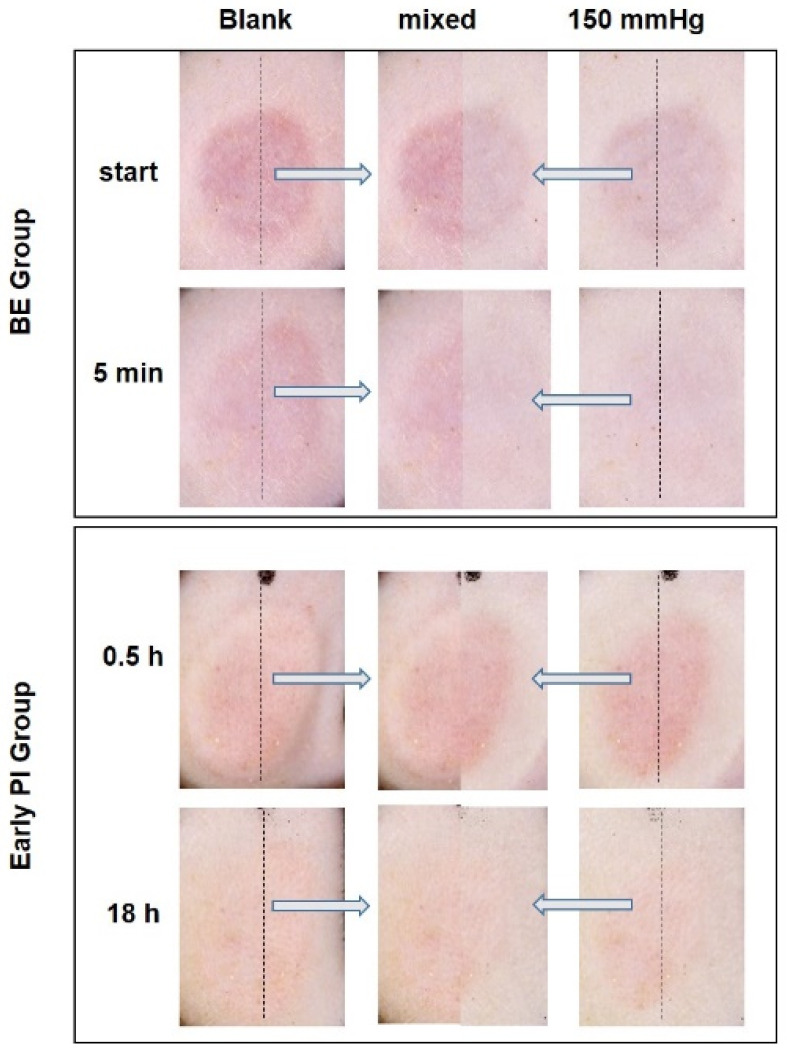
Observation of false positives and false negatives of erythema when the transparent disc was applied. Photographs taken by the Dermo-camera show the loaded area of the BE group at the start and 5 min after decompression before and after the use of transparent disc method. Blank (**left**) and 150 mmHg (**right**); merged pictures (**middle**). Pictures taken using the Dermo-camera showed the loaded area of the early PI group 0.5 and 18 h after decompression before and after the application of the transparent disc method. Blank (**left**) and 150 mmHg (**right**); merged pictures (**middle**).

After decompression, BE remained when the transparent disc was applied, and it was visually non-blanchable (Figure 5, top), although the a* value indicated that the degree of reduction in the level of blanching was obvious (Figure 2, bottom). By contrast, the erythema of the early PI was lightest at 18 h after decompression (Figure 4) decreased significantly when the transparent disc was applied, and was visually blanchable (Figure 5).

## 4. Discussion

The transparent disc method is an indicator of early PI diagnosis and is widely used in clinical settings [1]. However, in the present clinical diagnosis, no uniform standard is used for the pressure applied in the transparent disc method, and it varies from person to person. This condition significantly affects the results and consistency of clinical diagnosis [4]. In our preliminary study, we also found that the pressure applied in the transparent disc method varied from person to person according to their gender and body position (Supplemental Figure S1).

To explore the appropriate pressure to use in the transparent disc method, we performed tests by using BE models. The results obtained before and after pressure was applied showed that when the pressure was set at 50 mmHg, the erythema visibly faded in each period after decompression. Quantitative analysis showed that the gray values were statistically significant when the pressure was 100 mmHg. Close to the maximal values of fading were obtained when the pressure was 150 mmHg. Therefore, the pressure applied in the transparent disc method should be set at 50 mmHg (macroscopic observation and a* value) or 100 mmHg (gray value). The measurements of the gray value at the erythema and a* value of the spectrophotometer can demonstrate the degree of fading, and the a* value is more sensitive. It seems that the pressure observed to be used in the transparent disc method immediately after BE decompression was inappropriate, because the residual erythema was worse. There was obvious fading of BE within a short period of time. This finding can be applied for use in clinical observations.

Furthermore, we also constructed an early PI model. When applying a pressure of 150 mmHg to the skin, the erythema at each time period showed an obvious residual effect, which meets the definition of it being non-blanchable. This phenomenon was similar to that observed at 18 h after decompression, and the erythema was obviously weakened when pressed, similar to BE.

The transparent disc method was first used by dermatologists to distinguish skin hyperemia and hemorrhage [9], and later used for the early diagnosis of PI [10]. The observation methods used include the finger and transparent disk method. These can be performed by lightly pressing on the erythema area with either a finger or a transparent disc. If the erythema does not fade, the non-blanchable erythema seen in early PI is considered [11,12]. A previous study that compared the two methods found no obvious difference between the two transparent disc methods [4].

A diagnosis of early PI is made using the transparent disc method, which is widely used because of its simplicity. Nevertheless, the degree of light pressure that should be used has not yet been determined, which certainly affects the accuracy of this method. Therefore, whether early PI is included in the overall incidence rate has become an important issue that needs to be addressed [1]. Considering the omission of some epidemiological statistics, the prevalence and incidence rates are lower than the actual prevalence of PI. Kaltenthaler [5] calculated and compared the prevalence and incidence of PI in the UK, the USA and Canada, finding that many studies exclude early PI from their calculation, leading to significant differences in PI epidemiological statistics. The prevalence rates of PI were reported to be as low as 3% in Italy and Germany, while the rates observed in the UK and the Netherlands as high as 20%. A possible reason for this discrepancy may have been the inclusion of early PI in the statistics rather than the quality of nursing care [13]. This study revealed a high prevalence of pressure damage (18.6%) in contrast to the published DoH figure of 6.7% [14]. In this study, the authors ignored stage 1 damage, resulting in a prevalence of 10.1%. Considering the variations in the methodologies and the lack of consensus on a definition of pressure damage and the population surveyed, the standardization of the transparent disc method needs to be conducted urgently to determine the degree of light pressure to be applied.

Our study simulated the transparent disk method; the results obtained were in accordance with the definition of light pressure. However, macroscopic observations have some limitations. For example, the estimators cannot be used for comparison before and after and are also unreliable and hard to quantify [15]. Furthermore, we used a Dermo-camera to measure the gray values of the images and a spectrophotometer to measure the a* values. Based on previous clinical experience, the evaluator set the pressure to 150 mmHg for the transparent disc method [6]. In our animal experiments, the gray value and a* values showed that the pressure was in the range of 50–100 mmHg. In order to reduce secondary damage caused by inspections, pressures below 100 mmHg are more appropriate than 150 mmHg.

In human observations, the definition of light pressure has remarkable discrepancies from person to person. While the pressure applied varied from person to person, the pressure applied to most volunteers was below 20 mmHg, and our research results showed that it should be above 50 mmHg for the transparent disc method to be effective. We know that the blood hydrostatic pressure of peripheral circulation in the physiological state is 35 mmHg, and external pressure exceeding this will completely block the blood supply. However, the pathological state of early PI is different. In our preliminary experiment, the results showed that the a* value of the erythema was close to the lowest under a pressure of 150 mmHg, indicating that the transparent disc method has the best effect when observing PI erythema at a pressure close to 150 mmHg. At the same time, the vulnerability of early PI in the pathological state should be considered, and the pressure used in the transparent disc method should be reduced as much as possible. It is not known whether the rapid application of a pressure of 50–100 mmHg in a short time by the transparent disc method will also cause damage. A previous in vitro study showed that tissue exposed to 50 mmHg of pressure for 4 h still maintained a fiber bundle structure and capillaries of varying caliber. However, after being exposed to a pressure of 170 mmHg for 4 h, the tissue structure became significantly different [16]. This results demonstrated that the application of 50–100 mmHg of pressure may cause little to no harm.

The results obtained from the volunteers further indicated that the specific pressure used should be specified in order to prevent false negatives caused by the application of insufficient pressure, thereby affecting the diagnosis of early PI.

Nevertheless, the transparent disc method is limited to use in qualitative examinations and cannot meet clinical needs. It should be developed into a quantitative examination in order to evaluate cases of early PI more accurately in the future. The Dermo-camera can be used as an objective instrument in clinical examinations. We found that its use in combination with the transparent disc method is able to determine the pressure applied less effectively than the use of the spectrophotometer alone. Therefore, the Dermo-camera should be used to observe and record the fading of erythema under a specified level of pressure, further enhancing the accuracy of the pressure transparent disc method. Nevertheless, the Dermo-camera is an expensive device. Therefore, cheap devices need to be developed for use in the future.

In addition, some exceptions can be noted in our clinical observations. The erythema in early PI can be neither non-blanchable or blanchable, depending upon the severity of the disease [17], due to the overlapping histological changes seen in normal, blanchable and non-blanchable states [18]. This study also showed that the erythema seen in early PI was visually blanchable during the deterioration process. This phenomenon may be caused by a reduction in hyperemia and congestion. Similarly, BE shows visual non-blanchable changes, combined with an obvious reduction in and disappearance of erythema within a short period of time, which is consistent with the clinical manifestations of congestion and the results of our previous research [7], indicating that congestion may also occur in BE. Therefore, in addition to the routine use of the transparent disc method in clinical settings, if time permits, we also recommend observing the erythema at 5 min or 10 min after the first observation to compare the level of fading before and after the procedure. Our research showed that obvious fading should be placed in the category of congestion erythema. This setting requires the use of a corresponding recording device, it is difficult to achieve consistency solely by the evaluator’s memory.

However, in this experiment we mainly utilized animal models to simulate PI and carry out the transparent disc method. Our results may not reflect the PI situation in humans. Furthermore, we did not perform a pathologic analysis, this should be performed in further studies. We also need to set up a more rigid rule to distinguish the various degrees of symptoms in our further study.

Lastly, the Casio camera used in this study are devices designed for dermatologists for clinical observations. The price is high for clinical applications in places where PI is common, such as hospitals, nursing care facilities for the elderly, and home care. Therefore, a more convenient and cheaper device should be developed.

## 5. Conclusions

Our study showed that the appropriate pressure that should be used in the transparent disc method differs depending on the procedure used. Macroscopic and spectrophotometric observations were carried out at 50 mmHg, and gray values were measured by a Dermo-camera at 100 mmHg. For both BE and early PI, the results of the transparent disc method showed exceptions.

This is the first time the appropriate pressure that should be used in the transparent disc method has been validated; this study thus provides an objective index for the diagnosis of early PI.

## Data Availability

The data that support the findings of this study are available on request from the corresponding author. The data are not publicly available due to privacy or ethical restrictions.

## Supplementary Materials

The following supporting information can be downloaded at: https://www.mdpi.com/article/10.3390/diagnostics12051075/s1, Figure S1: Pressure of the finger method in human observation. 20 volunteers included in the study. They got the pressure when in the supine and lateral positions. Data are expressed as mean ± SEM.
