# Supplementary material for: Establishing an Appropriate Pressure for the Transparent Disc Method to Distinguish Early Pressure Injury and Blanchable Erythema
Source: Diagnostics (Basel). 2022 Apr 25;12(5):1075. doi: 10.3390/diagnostics12051075 (PMC9139210; doi:10.3390/diagnostics12051075)
Supplement: Supplementary file 1 [file diagnostics-12-01075-s001.zip › diagnostics-1635312-supplementary.pdf]

Human observation

This study was set in Yangzhou Medical College in China. A total of 20 volunteers participated in the study. They are 10 men and 10 women. They all studied medical knowledge systematically. All participants were healthy. Beforehand, the volunteers were explained the light pressure method of finger method according to the definition of early PI of NPIAP, and they were familiar with the operation of the instrument. The instrument used in the experiment was a digital force gauge (DST series, Imada Co., Japan). Volunteers pressed the attachment of the pressure device with finger slightly, and then recorded the applied pressure displayed on the screen of the instrument. During the experiment, the pressure device was placed vertically and horizontally to simulate supine and lateral positions for volunteers to press. The experiment was carried out in a quiet environment in order to avoiding errors. Pressure on the instrument was recorded in time.

All the participants provide an informed consent. Human observations were approved by the Ethics Committee of Yangzhou Medical College in 2020 (ethics approval number: YZUHL2021030). The registration number was ChiCTR2100052909 and the name of trial registry was the study of pressure ulcers.

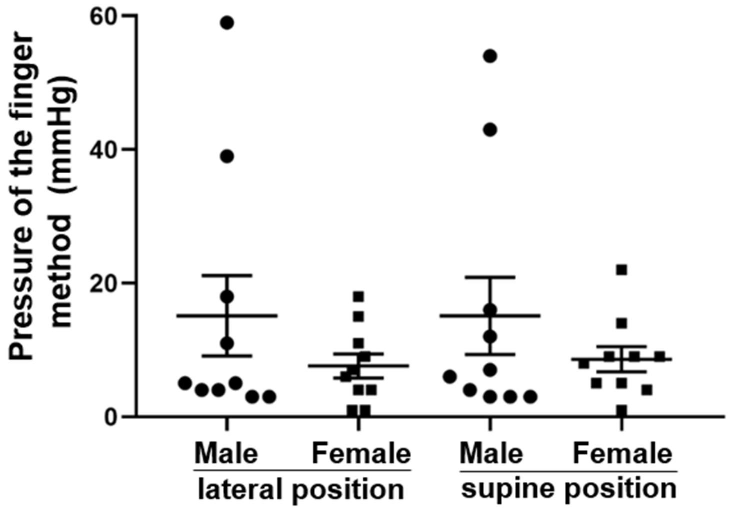

Supplemental Figure S1. Pressure of the finger method in human observation. 20 volunteers included in the study. They got the pressure when in the supine and lateral positions. Data are expressed as mean ± SEM.
